# P-2333. Evaluation of Clinical Characteristics, Risk Factors and Prognosis of Herpes Zoster (Shingles) Infection in Türkiye: VARICOMP-Adult-2 Study

**DOI:** 10.1093/ofid/ofae631.2485

**Published:** 2025-01-29

**Authors:** Ozge Ozgen-Top, Zehra Karacaer, Ece Firuze Ozkan, Hasan Selcuk Ozger, Nese Saltoglu, Nefise Oztoprak-Cuvalci, Ayse Seza Inal, Birsen Mutlu, Damla Boztas, Rehile Zengin, Alpay Azap, Sema Alp-Cavus, Ali Acar, Didem Tuba Akcali, Irem Akdemir, Dilek Bayramgurler, Hande Berk-Cam, Ahmet Cagkan Inkaya, Dilek Dasgin, Secil Deniz, Gamze Erfan, Ozlem Guler, Nilsel Ilter, Behice Kurtaran, Zekayi Kutlubay, Selda Sayin-Kutlu, Ayse Sesin Kocagoz, Ener Cagri Dinleyici, Esin T Senol

**Affiliations:** Gazi University Faculty of Medicine, Ankara, Ankara, Turkey; University of Health Sciences Gulhane Training and Research Hospital, Ankara, Ankara, Turkey; Pamukkale University Faculty of Medicine, Denizli, Denizli, Turkey; Gazi University Faculty of Medicine, Ankara, Ankara, Turkey; Istanbul University-Cerrahpasa, Cerrahpasa Faculty of Medicine, Istanbul, Istanbul, Turkey; University of Health Sciences Antalya Training and Research Hospital, Antalya, Antalya, Turkey; Cukurova University Faculty of Medicine, Adana, Adana, Turkey; Kocaeli University Faculty of Medicine, Kocaeli, Kocaeli, Turkey; Hacettepe University Faculty of Medicine, Ankara, Ankara, Turkey; Acibadem University Faculty of Medicine, Acibadem Altunizade Hospital, Istanbul, Istanbul, Turkey; Ankara University Faculty of Medicine, Ankara, Ankara, Turkey; Dokuz Eylul University Faculty of Medicine, Izmir, Izmir, Turkey; Atilim University Faculty of Medicine, Ankara, Ankara, Turkey; Gazi University Faculty of Medicine, Ankara, Ankara, Turkey; Ankara University Faculty of Medicine, Ankara, Ankara, Turkey; Kocaeli University Faculty of Medicine, Kocaeli, Kocaeli, Turkey; University of Health Sciences Antalya Training and Research Hospital, Antalya, Antalya, Turkey; Hacettepe University Faculty of Medicine, Ankara, Ankara, Turkey; Cukurova University Faculty of Medicine, Adana, Adana, Turkey; Pamukkale University Faculty of Medicine, Denizli, Denizli, Turkey; Acibadem University Faculty of Medicine, Acibadem Altunizade Hospital, Istanbul, Istanbul, Turkey; Kocaeli University Faculty of Medicine, Kocaeli, Kocaeli, Turkey; Gazi University Faculty of Medicine, Ankara, Ankara, Turkey; Cukurova University Faculty of Medicine, Adana, Adana, Turkey; Istanbul University-Cerrahpasa, Cerrahpasa Faculty of Medicine, Istanbul, Istanbul, Turkey; Pamukkale University Faculty of Medicine, Denizli, Denizli, Turkey; Acibadem University Faculty of Medicine, Acibadem Altunizade Hospital, Istanbul, Istanbul, Turkey; Eskisehir Osmangazi University Faculty of Medicine, Eskisehir, Eskisehir, Turkey; Gazi University Faculty of Medicine, Ankara, Ankara, Turkey

## Abstract

**Background:**

The varicella-zoster virus induces a primary infection known as varicella (chickenpox) and subsequently becomes latent in the dorsal root or cranial nerve ganglia. Later in life, the virus may reactivate, leading to a secondary infection called herpes zoster (shingles). Despite the increasing population at risk, research on shingles infection in Türkiye is limited. This study aimed to assess the clinical and demographic characteristics of shingles and the risk factors for postherpetic neuralgia among adults in Türkiye.

**Table 1.**
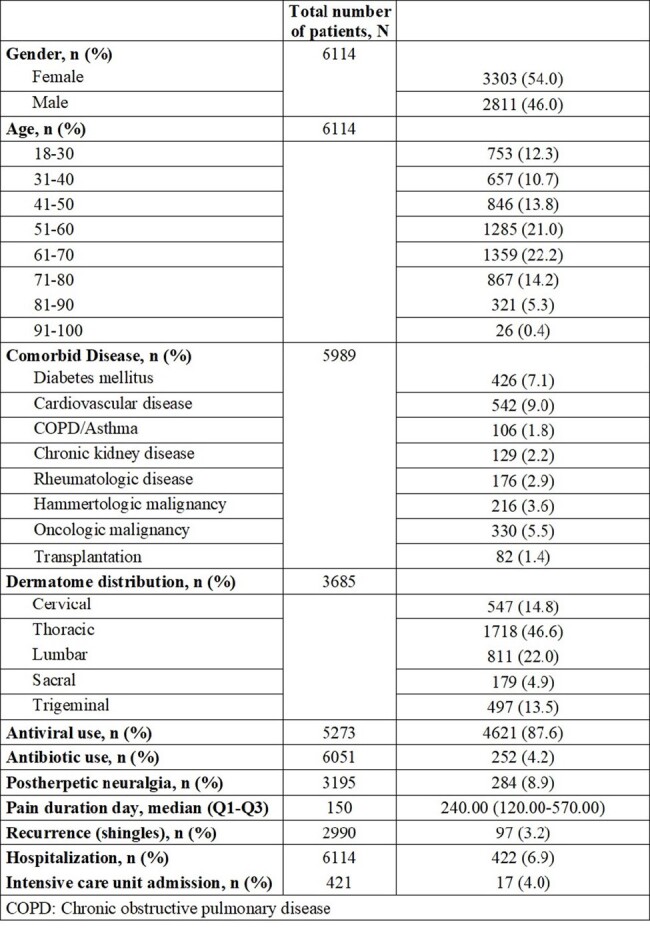
Demographic and clinical information of the patients

**Methods:**

This retrospective, descriptive, multicentre (n=11) study was conducted between January 2016 and January 2022. All patients aged ≥ 18 years who were diagnosed with shingles following screening based on ICD-10 codes related to these conditions were included. Logistic regression analysis was conducted to identify independent risk factors for postherpetic neuralgia.

**Table 2.**
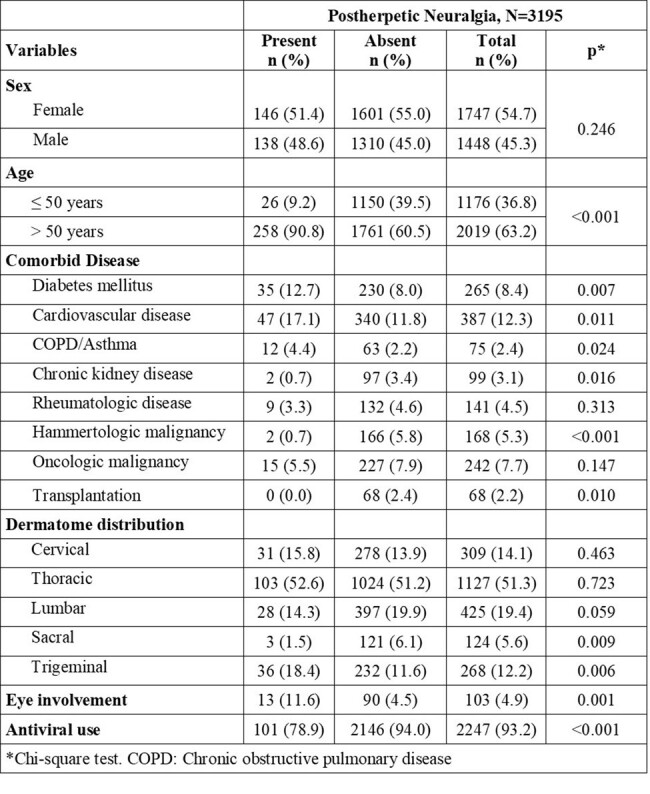
Comparative analyses regarding postherpetic neuralgia development of the patients

**Results:**

A total of 6114 patients with herpes zoster infection were evaluated. The demographic and clinical characteristics of the patients are presented in Tables 1 and 2. The age groups with the highest proportion of shingles were 61-70-year (22.2%, n=1359) and 51-60-year (21.0%, n=1285), respectively. The most prevalent comorbidities were cardiovascular diseases (9.0%, n=542) and diabetes mellitus (7.1%, n=426). Concerning dermatome distribution, the most affected region was the thoracic region (46.6%, n=1718). Significant independent risk factors for developing postherpetic neuralgia were being ≥ 50 years of age (OR=3.356, 95% CI, 1.795-6.274), trigeminal involvement (OR=2.496, 95% CI, 1.450-4.298), and antiviral use (OR=0.165, 95% CI, 0.094-0.290) (Figure1).

**Figure 1.**
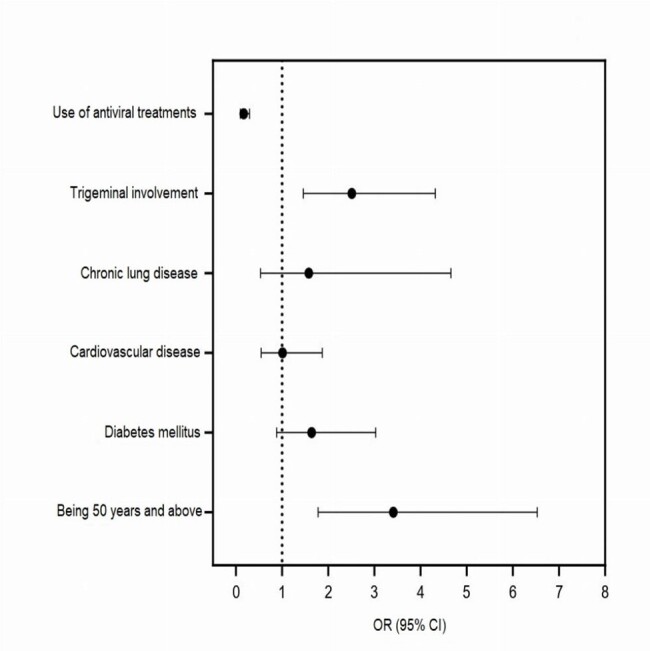
Risk factors for the development of postherpetic neuralgia. OR: Odds ratio; CI: Confidence interval.

**Conclusion:**

This study results revealed that shingles increase with advanced age and comorbidities. It is anticipated that the burden of shingles disease and related complications will increase due to the increasing aging population and comorbidities in Türkiye. Given the burden, especially in the ≥ 50 years age group, it underscores the importance of employing protective measures such as herpes zoster immunization and using antiviral treatments to prevent postherpetic neuralgia.

**Disclosures:**

Ozge Ozgen-Top, Asisst. Prof., MD, GSK Biologicals SA: Honoraria|GSK Biologicals SA: The study was funded by GSK Biologicals SA;GSK Biologicals SA reviewed a preliminary version of the abstract;authors have the final responsibility Esin T. Senol, Prof., MD, GSK Biologicals SA: Grant/Research Support|GSK Biologicals SA: Esin Şenol will receive honoraria for IDWeek 2024 if the abstract is accepted.

